# Value of hyperspectral data for wall to wall wetland vegetation mapping in heterogeneous landscapes

**DOI:** 10.1038/s41598-026-44275-0

**Published:** 2026-03-17

**Authors:** Anna Jarocińska, Dominik Kopeć, Jan Niedzielko, Anna Halladin-Dąbrowska, Marlena Kycko

**Affiliations:** 1 Faculty Of Geography and Regional Studies, University Of Warsaw, Krakowskie Przedmieście 30, 00-927 Warsaw, Poland; 2 Faculty of Biology and Environmental Protection, Department of Biogeography, Paleoecology and Nature Conservation, University of Lodz, Banacha 1/3, 90-237, Łódź, Poland; 3 MGGP Aero Sp. z o.o., Kaczkowskiego 6, 33-100, Tarnów, Poland

**Keywords:** Remote sensing, LiDAR, Narew National Park, Filed mapping, Texture features, Ecology, Ecology, Environmental sciences, Optics and photonics

## Abstract

Remote sensing is increasingly employed today for the creation of wall-to-wall vegetation maps of protected areas. The main objective of this study was to evaluate whether it is feasible to develop a remote sensing-based wall-to-wall vegetation map of wetlands that is useful for nature conservation purposes without hyperspectral data. The research was conducted in the Narew National Park in north-eastern Poland. In 2020, three types of airborne data covering an area of 325 km^2^ were acquired: hyperspectral, Light Detection and Ranging data (LiDAR), and from visible and near infrared range (RGBN) imagery. At the same time, botanists collected 1,921 reference polygons for training and validating the machine learning model. Then, classifications were conducted using the CatBoost algorithm with four different scenarios created from the mentioned datasets. The results indicate that, based on hyperspectral and LiDAR data, it is possible to produce a wall-to-wall vegetation map with an overall accuracy of 0.82. When hyperspectral data are replaced by less expensive and widely accessible RGBN data, accuracy declines by 0.10; this reduction decreases to 0.09 if textural features derived from the RGBN data are additionally incorporated into the classification. In scenarios where only LiDAR data are available, vegetation classification accuracy is much lower, reaching 0.63. Comparison of the remote sensing maps with a vegetation map created through field mapping methods indicates that, irrespective of whether hyperspectral or only RGBN and LiDAR data are available, remote sensing maps contribute substantial new knowledge regarding wetland vegetation in spatially heterogeneous landscapes and are highly useful for their conservation management.

**Supplementary Information:**

The online version contains supplementary material available at 10.1038/s41598-026-44275-0.

## Introduction

Remote sensing is currently one of the fundamental tools for wetland monitoring. It can be used as a method for analysing wetland desiccation^1^, identifying threats from biological invasions^2^, delineating wetland boundaries^3,4^, and producing comprehensive vegetation maps^5^. Remote sensing is applied as a monitoring tool at various scales, ranging from local scales encompassing individual small wetlands^6, 7^ through regional analyses^3^, to national^8^, continental^9^, and even global scales^10^. The data sources for these analyses are Unmanned Aerial Vehicle (UAV), airborne or satellite data, with particular emphasis on freely available Sentinel-2 and Landsat datasets due to their accessibility.

For wetland monitoring conducted at a local scale, airborne or UAV data can be an alternative to satellite data. Although they are less commonly used, an indisputable advantage of data acquired from altitudes lower than those of satellites is the higher spatial resolution (GSD ≤ 1 m) and the possibility of using data with greater spectral resolution. There are commercially available hyperspectral (HS) imaging scanners with several hundred bands from the Visible and Near Infrared (VNIR) and Short Wave Infrared (SWIR) spectral regions of the electromagnetic spectrum. The higher spectral resolution and the broad VNIR-SWIR range of hyperspectral imaging are related to more effective identification of vegetation types. Studies carried out in Natura 2000 habitats in Poland^11^ demonstrated an average 0.14 increase in classification accuracy using HS data compared to Sentinel-2 images. The effectiveness of hyperspectral data for emergent wetland vegetation classification has been demonstrated across diverse wetland ecosystems, including lake environments^12^. Another advantage of airborne data is the possibility of sensor fusion (e.g., HS and LiDAR), which improves classification accuracy^13^. Moreover, many wetland types exhibit fine‑scale spatial heterogeneity and complex vegetation patterns, so the use of high‑resolution airborne or UAV data is particularly advantageous^14–16^. Recent scientific publications demonstrate that the fusion of Airborne Laser Scanning (ALS) and HS data is the optimal source of information for wetland vegetation classification, enabling high classification accuracy^12,17,18^. Advanced machine learning approaches, including ensemble methods and deep learning networks, have been developed to optimize the integration of these multisource datasets, with feature enhancement and fusion networks showing particular promise for coastal wetland classification^19,20^. The application of these techniques extends to diverse wetland environments, from karst wetlands^21^ to Arctic ecosystems^22^, demonstrating the versatility of hyperspectral-LiDAR fusion approaches.

Maps derived from traditional field-based vegetation mapping following the Braun-Blanquet approach^23^ are widely used for monitoring natural environments. However, such maps are based on discrete field samples^24^, from which vegetation patterns are interpolated across unsampled areas. In contrast, approaches combining remote sensing data and machine learning rely on spatially continuous information and integrate multiple data-derived features to delineate vegetation patches across the landscape.

Direct comparisons of vegetation maps derived from field-based mapping and remote sensing analyses conducted for the same study area and at comparable spatial scales are still limited^25,26^. The application of remote sensing techniques enabled the identification of a greater number of vegetation class patches^27^. Recent developments in automated tools, such as the NaturaSat software system, facilitate the identification, monitoring, and assessment of habitats using remote sensing techniques, bridging the gap between field-based and remote sensing approaches^27^. On the other hand, not all information is discernible from image data^28^. The use of vegetation maps generated through remote sensing and machine learning (ML for the management of protected areas requires high accuracy of the classification,thus, the use of airborne HS and LiDAR data seems to be the best solution^15,16,22,29^. Furthermore, the informativeness of these data can be enhanced with transformations like feature extraction methods like Minimum Noise Fraction (MNF) are performed to remove noise and reduce data volume^30^. The LiDAR data can be used to calculate a number of features describing the structure of vegetation, including three-dimensional structural metrics that capture vertical heterogeneity and canopy complexity^15,16,31^.

Another way to improve the classification results is calculate texture features, which can be acquired from LiDAR^15,16^ LiDAR and HS^32^, and RGBN data^33^. These data provide statistical information on the group-of-pixels level. One of the most commonly used tools for deriving texture features currently is the Haralick method^34^. Texture features extracted from remote sensing data provide additional information on the spatial arrangement and surface variability. By capturing fine‑scale heterogeneity, texture metrics improve the differentiation of diverse vegetation types in structurally complex open wetland habitats, thereby enhancing classification performance. The utility of texture layers has been demonstrated for diverse vegetation types, e.g.: aquatic vegetation^35^, woody vegetation in a rural area^36^ plain and mountainous environments or crops^37^. It is particularly useful for delineation of the patches^32,38^.

A significant limitation of airborne HS data is the relatively high acquisition cost, which is not always affordable for protected area managers. Consequently, there is a need for more cost-effective alternatives to HS data combined with ML tools that can achieve comparable accuracy while maintaining spatial resolution at or below 1 m. One research direction is using LiDAR data only for vegetation identification. However, the informativeness of LiDAR is insufficient to produce reliable vegetation maps, the overall accuracy of such maps is around 0.50^39^. The same unsatisfactory results are mostly achieved when only RGB and RGB-NIR data are used in vegetation classification^40,41^. These data are easy to acquire with very high spatial resolution, enabling the acquisition of texture features. The multisensor fusion of RGB/RGBN data, including texture layers and LiDAR data, represents a potential alternative to HS data. Previous studies with this data combination have been conducted, for instance, in China for the classification of vegetation types in open-pit mining regions, achieving an overall accuracy (OA) of 0.98^42^. The data fusion of LiDAR and RGBN data is quite popular and is considered an accessible airborne data combination. However, its application in wetland vegetation mapping remains insufficiently investigated.

The present study aimed to answer the following research questions:

1. Is there a significant decrease in wetland vegetation classification accuracy when replacing HS with RGBN data?
2. Does the inclusion of texture features derived from RGBN data in the classification process significantly improve the accuracy of the resulting map?
3. Whether wall-to-wall remote sensing vegetation maps brings additional knowledge, compared to maps made using traditional field mapping, useful for wetland conservation management

## Study area

**Fig. 1:**
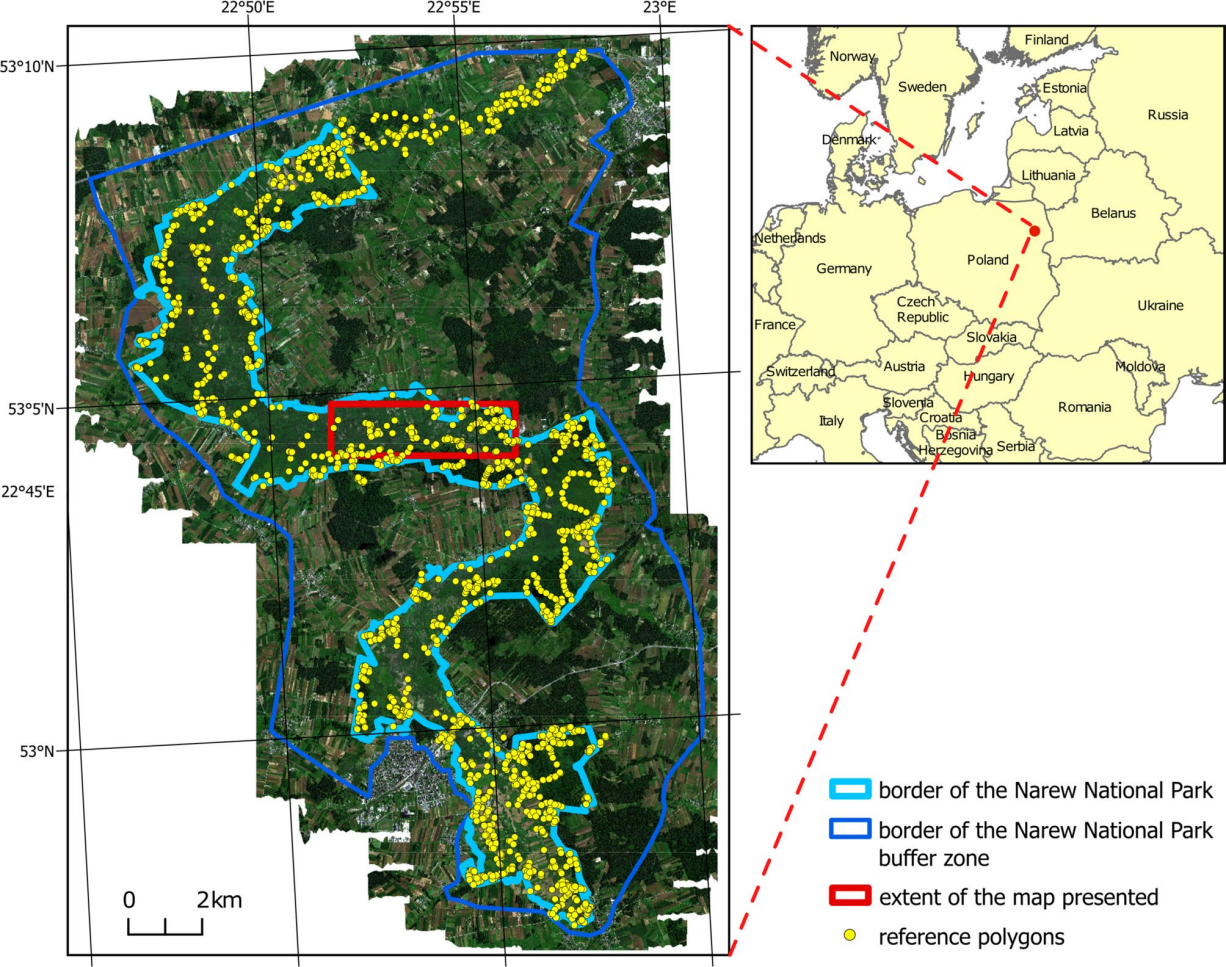
Map of the research area with its location in Europe.

The research was conducted within the boundaries of the Narew National Park (NNP) and in the northern part of its buffer zone (Fig. 1). NNP is simultaneously designated as a Special Area of Conservation under the Natura 2000 network (named ‘Narew Marshes’, unique area code PLH200002), a Special Protection Area for birds (named ‘Narew Marshland Valley’, unique area code—PLB200001), and a wetland site of international importance under the Ramsar Convention^43^. NNP is located in northeastern Poland, (Fig. 1). Geographically, the National Park lies within the Upper Narew Valley mesoregion, which forms part of the broader North Podlasie Lowland macroregion^44^.

According to the climatic regionalisation of Podlaskie Voivodeship, NNP is situated in the Podlasie climatic region, specifically within the Białystok subregion^45^. The area is characterised by a temperate transitional climate with pronounced continental influences, including harsh winters and warmer, drier summers relative to other parts of Poland. In 2022, the mean annual air temperature was 8.2 °C. The mean annual precipitation for the same year was approximately 550–600 mm^46^.

Narew National Park was established in 1996 and currently covers an area of approximately 6,810 hectares. Nearly the entire area (95%) consists of wetlands, situated on the riverine floodplain of the Narew River valley between the places of Suraż and Rzędziany^47^. Within the boundaries of NNP, the Narew River valley forms a flat, longitudinally oriented floodplain of variable width. The valley floor exhibits a very low gradient, approximately 0.19‰, and is diversified by small dune formations and a network of anastomosing river channels^48^. It is precisely this anastomosing channel system that imparts a unique character to this section of the Narew, making it one of the most distinctive fluvial landscapes in Europe. At the same time, it constitutes one of the largest floodplain wetland complexes in Europe. The present landforms are the result of both glacial processes and subsequent erosional and depositional activity. The valley is infilled with peat deposits that lie on shallow layers of silt, clay, or directly on sands^49^. The anastomosing character of the Narew River in this section is not solely the product of natural conditions. To a significant extent, it has a cultural origin: centuries of human intervention, including the construction of various hydraulic structures for milling and traditional fisheries, have played a critical role in shaping the river’s multi-channel pattern and enhancing the waterlogged character of the valley^50^.

The hygrophilous vegetation, which constitutes the dominant vegetation type within Narew National Park, has been shaped not only by prevailing hydrological conditions such as the source of wetland water supply, the spatial extent, timing, magnitude, and duration of inundation by floodwaters but also by land use practices, including the frequency and method of utilization, or complete abandonment thereof. These are primarily plant communities associated with lowland fens and other types of fluvially fed wetlands, periodically inundated by riverine waters covering the valley floor. The most widespread vegetation type in NNP consists of extensive reed beds (*Phragmitetum australis*). Also commonly encountered are grass- and sedge-dominated marshes, particularly *Glycerietum maximae* and *Phalaridetum arundinaceae* communities. Large areas are also occupied by meadow-like tall-sedge communities, dominated especially by *Carex gracilis*. Additionally, riparian forests, alder carrs, and shrubby formations are well represented. Other plant community types, such as meadow and grassland communities, tall herb communities (*Convolvuletalia*, *Filipendulion*), *calcareous spring-fed fens* (*Caricetalia davallianae*), moss-sedge communities belonging to the *Scheuchzerio-Caricetea nigrae* class, mudflat vegetation, and other forest types, occur much more sporadically. These are typically restricted to the marginal zones of the valley, particularly in the outer parts of broad basin sections, on elevated mineral hummocks, and in the southern section of the valley^51^.

In recent decades, NNP has experienced increasingly pronounced and adverse transformations in vegetation structure and composition. These changes are largely driven by the cessation of traditional agricultural use, especially on privately owned lands, and by substantial alterations in hydrological regimes. These include a weakening of flood dynamics and a progressive lowering of the groundwater table^52^. Consequently, there is a growing need for more frequent monitoring of vegetation status. However, given the vast area of the park and the inaccessibility of many wetland sites, such monitoring is virtually unfeasible using traditional field-based inventory and survey methods. In this context, remote sensing techniques offer valuable support and an effective alternative for long-term vegetation monitoring.

## Method

**Fig. 2:**
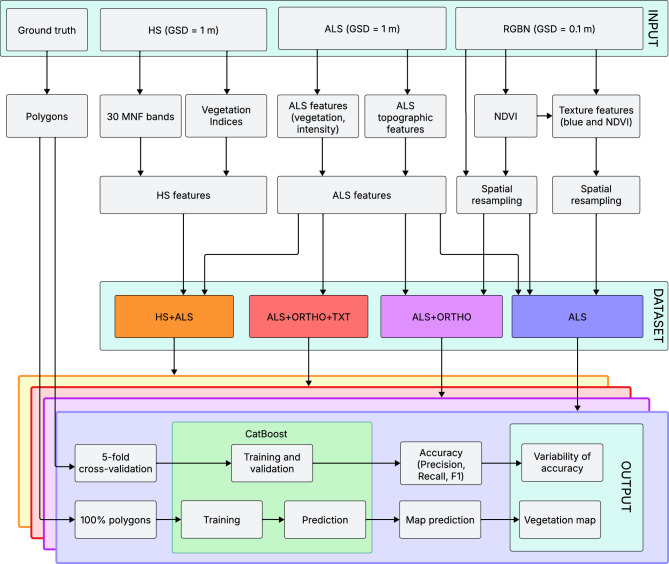
Overview of the data processing and classification workflow, including feature extraction from hyperspectral, ALS, and ortophotomap, dataset construction, and model evaluation using CatBoost with cross-validation. GSD—ground sampling distance, HS—hyperspectral, ALS—airborne laser scanning, ORTHO—RGBN ortophotomap, MNF—minimum noise fraction, NDVI—normalized difference vegetation index, TXT—texture layers.

The study was conducted in a series of successive stages (Fig. 2). In the initial stage, both aerial and ground reference data were acquired. Subsequently, the collected data were processed, and classifications were performed under four different scenarios, each accompanied by parallel evaluation procedures. Vegetation maps were then generated, and a statistical analysis was carried out to compare the results obtained across the different scenarios.

### Airborne data

The aerial data were acquired in two campaigns: hyperspectral and ALS data on 15th and 16th July 2020, and high-resolution multispectral camera (RGBN) on 18th July 2020. The area of the overflight was more than 325 km^2^, and the imagery covered the area of the NNP, including the buffer zone. The first overflight was conducted using the multisensor platform with three scanners: one laser and two hyperspectral scanners. Data were acquired in 58 flightlines. The ALS data were collected using a RIEGL LiDAR VQ-780II scanner with an average point cloud density of 7.6 points/m^2^^53^. The hyperspectral data were collected with 1 m spatial resolution using three HySpex scanners from NEO: one VNIR-1800 covering 182 bands in 0.4–0.9 μm and two SWIR-384 covering 288 bands in 0.9–2.5 μm (to increase spatial resolution in SWIR)^54^. Details regarding the platform and equipment can be found in previous works^32,55^. Second overflight was performed using a high-resolution PhaseONE iXU-RS1000 multispectral camera with four spectral bands: blue, green, red and NIR.

### Reference data collection for vegetation classification

Field-based reference data for vegetation classification, used for both training and validation purposes, were collected by botanists between July 15 and August 15, 2020. A mobile mapping application based on the Android operating system was employed to record geographic coordinates and attribute data for individual sampling points. Data collection was carried out using a Trimble DA1 external antenna, providing positional accuracy of up to 1 m. Each measurement involved georeferencing the centre of a circular plot using a GNSS receiver integrated into a mobile device and connected to the antenna. The minimum plot radius was 3 m; in the case of small or narrow vegetation patches, this was reduced to 2 m, and in exceptional situations to 1 m.

Reference polygons were established in sites that were both representative and typical of the vegetation community, and homogeneous in terms of species composition and vegetation structure. Sampling was explicitly avoided in ecotonal zones to minimise edge effects. All major vegetation types occurring within the study area including forest and non-forest, aquatic and terrestrial communities were included. Vegetation was identified at the most detailed possible syntaxonomic level.

The spatial distribution of sampling points was designed to reflect the actual spatial heterogeneity of vegetation across the area of the NNP, considering the occurrence of plant communities within individual image acquisition blocks and flight lines. The number of reference points for each community was proportional to its areal extent within the study area and accounted for internal floristic and structural variability.

All field-based reference data underwent quantitative and qualitative validation procedures. In total, 1,789 sampling points were collected during the field campaign, corresponding to 115 distinct vegetation units at various levels of the syntaxonomic hierarchy. These units were then aggregated into 28 broader classes for use in the classification process (Table1). In order to determine a minimum of 20 reference polygons representing each class, small classes were combined taking into account the syntaxonomic hierarchy and spectral similarity. Only spectrally similar classes whose merging was also justified from a phytosociological perspective were combined. Vegetation units representing syntaxonomically distant plant communities were deliberately excluded from aggregation. In addition, 132 reference polygons were generated during post-fieldwork analysis to represent surface water bodies and areas characterised by various types of non-vegetated land cover. For classification result analysis, individual vegetation classes were grouped into broader categories of plant community types. The aggregation of legend units was based on phytosociological, morphological, and functional criteria, taking into account similarities in vegetation structure, land-use patterns, species composition, and the ecological roles of the studied communities. Table 1 provides an overview of the vegetation classes, the aggregated class groups resulting from the merging process, and the number of reference polygons and pixels assigned to each class for supervised classification.

**Table 1:** The classes used in the analysis with the division of different vegetation types, the number of reference polygons and pixels calculated for 1 m spatial resolution (px).

No	Class label (full)	Legend label	No. of polygons	No. of px
Macrophyte vegetation				
1	Free-floating and rooted macrophyte vegetation (*Potamogetonetea, Lemnetea*)	*Potamogetonetea, Lemnetea*	95	1742
Helophyte communities				
2	Sedge-dominated swamp communities (*Magnocaricion*)	Sedge swamps	103	2396
3	Reed canary grass stands (*Phalaridetum arundinaceae*)	*Phalaridetum arundinaceae*	95	2483
4	Mannagrass swamp community (*Glycerietum maximae*)	*Glycerietum maximae*	46	1106
5	Cattail marshes* (Typhetum angustifoliae, Typhetum latifoliae)*	*Typhetum angustifoliae and T. latifoliae*	26	439
6	Common reed beds (*Phragmitetum australis*)	*Phragmitetum*	86	2406
7	Other tall helophyte communities (*Phragmito-Magnocaricetea*)	Other *Phragmito-Magnocaricetea* comm	64	1059
Lowland fens				
8	Lowland fens and transitional mires (*Scheuchzerio-Caricetea*)	*Scheuchzerio-Caricetea*	44	1044
Grasslands				
9	Dry grasslands on sandy soils (*Koelerio-Corynephoretea*)	*Koelerio-Corynephoretea*	61	972
10	Nardus grasslands and heathlands (*Nardetalia strictae, Vaccinio myrtilli-Genistetalia pilosae*)	*Nardetalia, Vaccinio-Genistetalia*	41	679
11	Low swards in periodically flooded or waterlogged locations (*Potentillo-Polygonetalia avicularis*)	*Potentillo-Polygonetalia*	51	852
12	Purple moor-grass meadows (*Molinion*)	*Molinion*	26	636
13	Floodplain and wet meadows (alliances *Alopecurion, Cnidion, Calthion*)	Other *Molinietalia* comm	140	3611
14	Mesic hay meadows and pastures (*Arrhenatheretalia*)	*Arrhenatheretalia*	143	4135
Tall-herb vegetation				
15	Tall-herb communities (*Filipendulion*)	*Filipendulion*	97	2425
16	Riparian tall herb fringe communities (*Convolvuletalia*)	*Convolvuletalia*	145	3342
17	Small-reed dominated grassland (*Calamagrostietum epigeji*)	*Calamagrostietum epigeji*	26	578
18	Creeping thistle—dominated community (*Cirsium arvense*)	Comm. *Cirsium arvense*	32	609
Willow thickets				
19	Rosemary-leaved willow-dominated community (*Salix rosmarinifolia*)	Comm. *Salix rosmarinifolia*	22	473
20	Mixed willow thickets (*Salicetum pentandro-cinereae*)	*Salicetum pentandro-cinereae*	28	1862
21	Riparian willow communities (*Salicion albae*)	*Salicion albae*	44	2379
Deciduous and coniferous forest				
22	Riparian forests and alder carrs (alliances *Fraxino-Quercion roboris, Alnion glutinosae*)	Riparian/alder for	122	9817
23	Mixed deciduous forests (*Tilio-Carpinetum*) and thermophilous oak woodlands (*Potentillo-Quercetum*)	Mixed/oak forests	27	7193
24	Coniferous forests (*Vaccinio-Piceetea*)	*Vaccinio-Piceetea*	26	2039
25	Birch-dominated community (*Betula* spp.)	Comm. *Betula* spp.	55	1105
26	Aspen-dominated community (*Populus tremula*)	Comm. *Populus tremula*	32	810
Other classes				
27	Mown or grazed grasslands	Mown or grazed grass	45	1187
28	Other non-forest vegetation types	Other non-forest comm	67	1491
29	Surface waters	Surface waters	57	2240
30	Other non-vegetated areas	Bare/non-veg. area	75	1708
Total no. of reference polygons/pixels			1921	62 818

### Raster data preprocessing

The ALS point cloud orientation was performed using RiProcess software (RiALITY^56^), then a point cloud was generated in RiAnalyze software (RiALITY^57^). The classification was done automatically in TerraSolid software^58^ and then manually according to ASPRS standard classes (LAS 1.4 Point Data Record Format 3). Details can be found in previous works^32,59^.The ALS point cloud was classified, and then raster products were generated: Canopy Height Model and statistical metrics in 1 m spatial grid cells describing vegetation properties such as height, reflectance, and density. Additionally, based on the generated Digital Terrain Model topographic features were acquired in SAGA software. Details regarding ALS features calculation can be found in a previous study^32^.

Hyperspectral images’ geometric processing was performed in PARGE (ReSe^60^), including parametric geocoding, orthorectification based on Digital Surface Model and conversion to at-sensor radiance (W·nm^−1^·sr^−1^·m^−2^). Data from VNIR and SWIR scanners were geometrically merged into one hyperspectral cube with the split wavelength at 935 nm. Atmospheric compensation was performed using ATCOR4 software (Rese^61^). Bands with wavelengths higher than 2.35 μm were removed due to excessive noise. A Savitzky–Golay filter with a 6-band window was applied to smooth the spectra. Finally, the images were then mosaicked into one hyperspectral image covering the study area. Then, a Minimum Noise Fraction transformation was performed, and the first 30 bands were selected. The selection of this number of bands was justified by previous studies, where the best classification results were achieved using this value^30^. To achieve the best classification results the spectral indices were calculated in ENVI 5.5^62^. Similar data were used in previous studies^32^. Details of the hyperspectral data processing can be found in other studies^55^.

The bands from the multispectral camera were used to produce the orthophotomap with a spatial resolution of 10 cm using INPHO OrthoVista (AMIGO^63^). To highlight the differences within the vegetation classes, the Normalised Difference Vegetation Index (NDVI) was calculated based on the normalised difference of NIR and RED.

The texture features were calculated in QGIS 3.14.10 using Orfeo Toolbox^64^. The analyses were performed based on blue and NDVI. These two bands have proved to be the most informative based on the results of experiments. Eight texture features were calculated using Simple Haralic Texture Extraction tool: Energy (texture uniformity), Entropy (measure of randomness of intensity image), Correlation (how correlated a pixel is to its neighborhood), Inverse Difference Moment (measures the texture homogeneity), Inertia (intensity contrast between a pixel and its neighbourhood), Cluster Shade, Cluster Prominence and Haralick Correlation. Features were calculated using a 3× 3 pixel kernel based on literature^65,66^. The equation can be found in Supplementary Material.

### Datasets

To test the utility of orthophotomap and the calculated texture, four datasets were created: one as a base and three to test the utility. The texture data were resampled in QGIS to 1-m spatial resolution using the average value. Then the four classification scenarios were created (Table 2). The HS-ALS was proven to be useful in previous studies, which is why this data combination was defined as the baseline set with the highest possible accuracy. The ALS products were accounted for in the second dataset to analyse the accuracy based only on one sensor. For the third dataset, ALS features were combined with four orthophotomaps and NDVI bands. This is one of the most commonly acquired remote sensing datasets^67,68^. The final one was enlarged with textural features counted from the blue band and NDVI. The difference between the used datasets relates not only to the method of acquisition and pre-processing, but also to the number of bands and volume. Fewer layers used in classification speed up the classification process.

**Table 2:** Number of features used in the four classification scenarios. For complete list of features see supplementary material.

Dataset name	Data	Number of features
HS-ALS	Base dataset: ALS features and MNF bands and spectral indices	152
ALS	ALS features	71
ALS-ORTHO	ALS features, four bands of orthophotomaps and NDVI calculated from ortophotomap	76
ALS-ORTHO-TXT	ALS features, four bands of orthophotomaps, NDVI calculated from ortophotomap and eight texture layers calculated based on blue and NDVI orthophotomap	92

### Classification

To test the utility of the datasets for vegetation identification, a classification was conducted. The CatBoost classifier was used for this process: classification and cross-validation were performed on reference polygons to assess the accuracy. The classifier was chosen based on previous results^32^. The accuracy assessment was performed using the fivefold cross-validation method. The reference dataset was divided into five parts. In each of the five iterations, 4/5 of the dataset was used for training, while 1/5 was used for validation. Classification accuracy was determined using confusion matrix, the accuracy for each class was analysed based on precision, recall, and the F1-score for each class, which were then averaged from fivefold cross-validation. The accuracy of different classification scenarios was compared based on these statistical parameters. The final step was the visualisation of the results. For each scenario, a classification map was generated based on the model trained on 100% of the reference dataset. The extent of the map presented is marked on the study area map (Fig. 1). When discussing the classification accuracy, the value of 0.8 was considered the optimal value, which is assumed in maps such as land cover (Global Dynamic Land^69^). However, due to the specificity and large number of classes, lower accuracy also seems to be acceptable for analysis.

### Map comparison

The next stage was to compare the maps for the four scenarios. The aim was to determine how the classification results differed not only at the reference polygon location but also beyond. In addition to comparing the distribution of classes over the entire classified section, three larger-scale areas were selected so that the similarity of the boundaries could be determined in detail. In addition to the qualitative analysis, a quantitative analysis was carried out. For this purpose, the Jaccard index was used, which determines the similarity between two sets based on dividing the intersection size by the union. The analysis was carried out on grouped classes. The area within each vegetation group was summed, and then for each combination of classification images, the indices for each group separately and the combined index for the vegetation classes were calculated. Four classes were excluded from this analysis: surface waters, bare or non-vegetated areas, mowed and grazed grass and other non-forest communities, which cover too small an area to be detected.

## Results

**Fig. 3:**
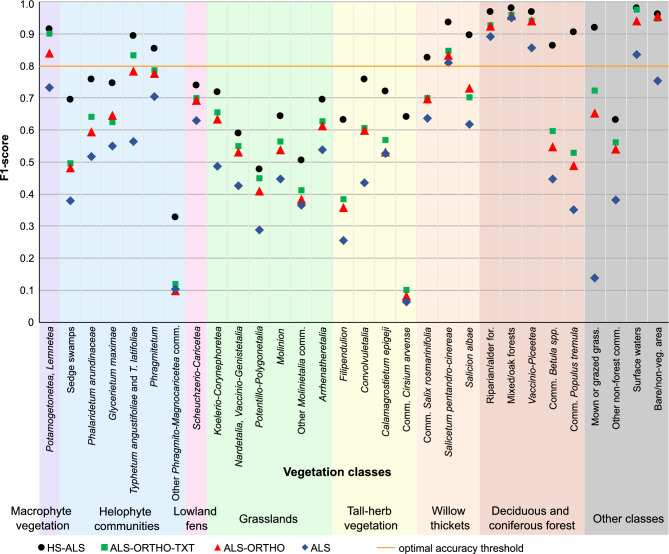
F1 score classification accuracy values for each class. Accuracy is reported as the average from fivefold cross-validation. For each vegetation class, four accuracy results from different scenarios (HS-ALS; ALS-ORTHO-TXT; ALS-ORTHO; ALS) are shown. Different broader vegetation groups are indicated by varying background colours. HS—hyperspectral, ALS—airborne laser scanning, ORTHO – RGBN ortophotomap, TXT—texture layers.

**Fig. 4:**
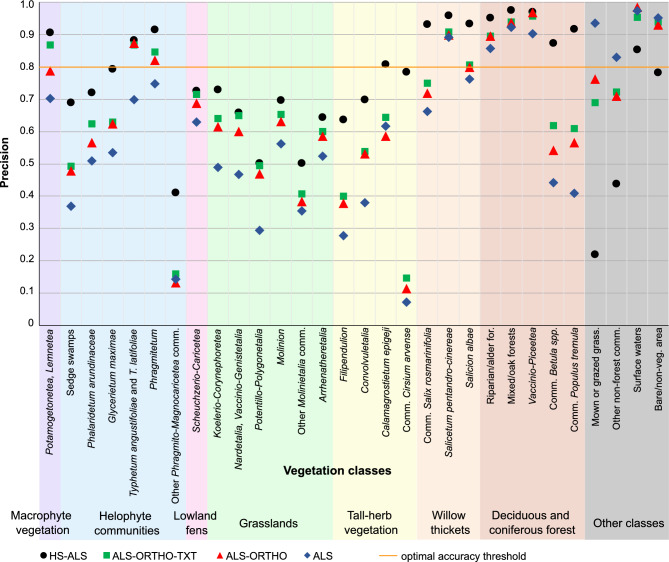
Precision values obtained for each class. Accuracy is reported as the average from fivefold cross-validation. For each vegetation class, four accuracy results from different scenarios (HS-ALS; ALS-ORTHO-TXT; ALS-ORTHO; ALS) are shown. Different broader vegetation groups are indicated by varying background colours. HS—hyperspectral, ALS—airborne laser scanning, ORTHO – RGBN ortophotomap, TXT—texture layers.

**Fig. 5:**
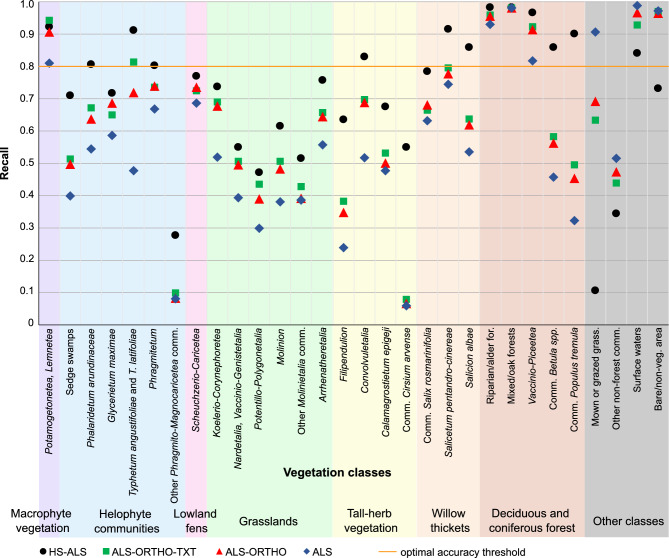
Recall values obtained for each class. Accuracy is reported as the average from fivefold cross-validation. For each vegetation class, four accuracy results from different scenarios (HS-ALS; ALS-ORTHO-TXT; ALS-ORTHO; ALS) are shown. Different broader vegetation groups are indicated by varying background colours. HS—hyperspectral, ALS—airborne laser scanning, ORTHO – RGBN ortophotomap, TXT—texture layers.

**Fig. 6:**
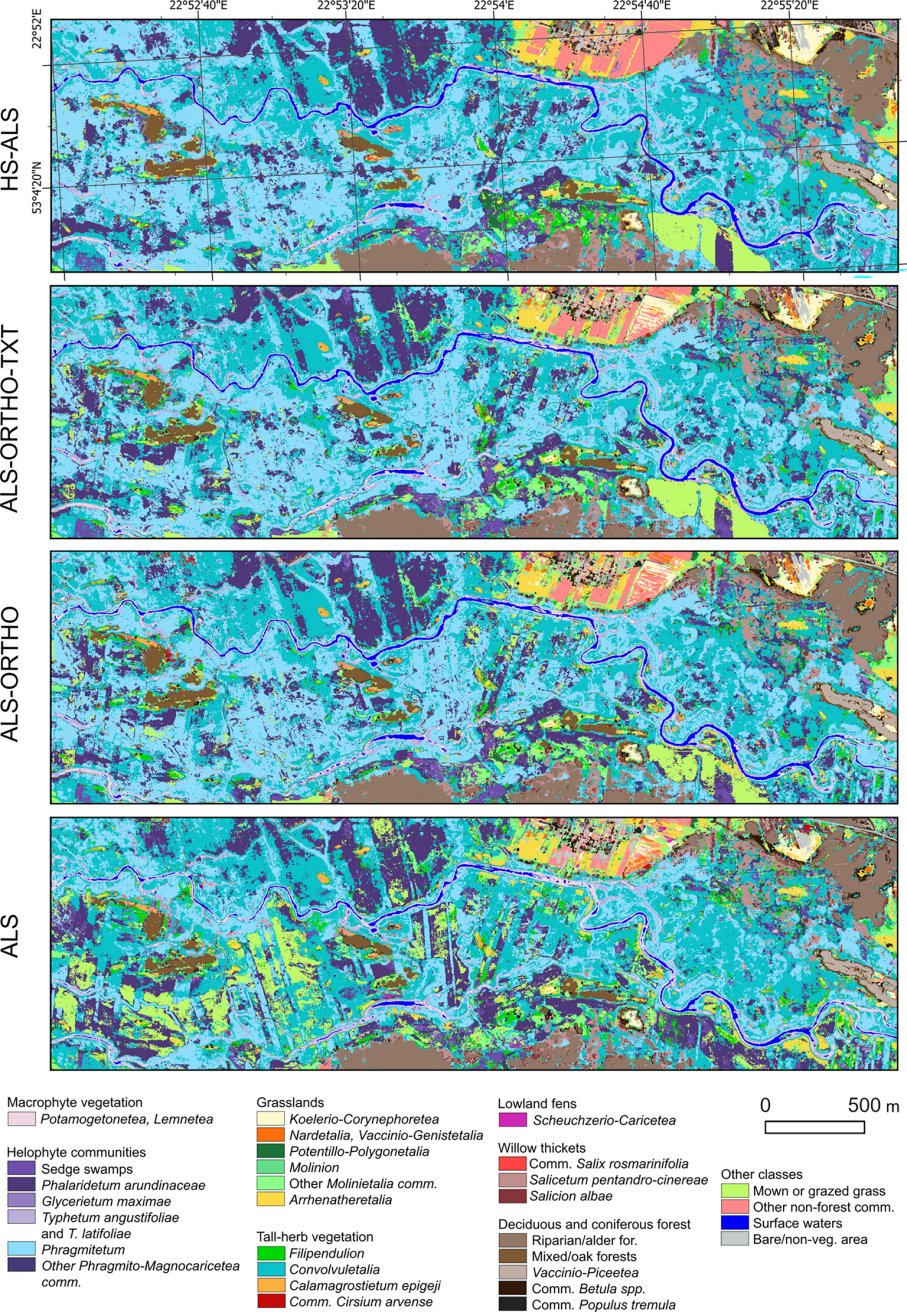
A section of the wetland vegetation map of Narew National Park, generated based on predictions from four models that differ in the input remote sensing datasets used (HS-ALS; ALS-ORTHO-TXT; ALS-ORTHO; ALS). In Fig. 1, the red frame indicates the part of the study area for which the map was generated. HS—Hyperspectral, ALS—airborne laser scanning, ORTHO – RGBN ortophotomap, TXT—texture layers.

The comparison of accuracy results across the four analyzed scenarios indicates that the model constructed using hyperspectral and airborne LiDAR data (HS-ALS) achieved the highest mean OA of 0.82 based on fivefold cross-validation (Fig. 3, see supplementary material). The second highest overall accuracy was obtained by the ALS-ORTHO-TEX scenario, reaching 0.73, followed closely by the ALS-ORTHO scenario with an OA of 0.72. The lowest accuracy metrics were recorded for the model using only ALS data, with an OA of 0.63. The HS-ALS scenario also demonstrated the highest average F1-score for the 30 distinguished classes, with a mean value of 0.77; 14 of these classes were with an F1-score ≥ 0.80. For each analyzed class, the highest Precision values were the highest for the HS-ALS scenario (Fig. 4). Regarding Recall, HS-ALS achieved the highest values for 29 out of 30 classes (Fig. 5). The only exception was the *Potametea* and *Lemnetea* classes, for which the highest Recall was observed under the ALS-ORTHO-TEX model. The models developed using ALS-ORTHO-TEX and ALS-ORTHO data yielded very similar mean F1-scores for the classes, equal from 0.65 to 0.63, respectively, with 8 and 7 classes achieving F1-scores ≥ 0.80. Significantly lower mean F1 (0.52) was achieved by the model performed on ALS data, with only five classes reaching an F1-score ≥ 0.80. This scenario also produced the lowest F1 values across almost all classes, the lowest Recall scores for 27 out of 30 classes, and the lowest Precision for 25 out of 30 classes. Pairwise analysis of map similarity indicated the highest congruence between maps generated using ALS-ORTHO-TEX and ALS-ORTHO data, with a Jaccard index of 0.92 across all classes (Fig. 6, Table 3). The lowest similarity was observed between the HS-ALS and ALS-ORTHO scenarios, with a Jaccard index of 0.68. Notably, a relatively high spatial similarity (0.82) was recorded between vegetation maps derived from models based on HS-ALS and ALS-ORTHO-TEX data.

**Table 3:** Map similarity determined by the Jaccard coefficient. Similarity was assessed based on the maps shown in Fig. 6.

Class	HS-ALS vs. ALS-ORTHO-TXT	HS-ALS vs. ALS-ORTHO	HS-ALS vs. ALS	ALS-ORTHO-TXT vs. ALS-ORTHO	ALS-ORTHO-TXT vs. ALS	ALS-ORTHO vs. ALS
All classes	0.82	0.68	0.81	0.92	0.75	0.76
Forests	0.85	0.81	0.85	0.93	0.88	0.88
Grasslands	0.61	0.40	0.59	0.76	0.49	0.52
Tall-herb Vegetation	0.60	0.43	0.60	0.80	0.52	0.53
Helophyte	0.75	0.56	0.74	0.88	0.64	0.65
Macrophyte	0.80	0.73	0.78	0.86	0.77	0.81
Willow thickets	0.49	0.41	0.50	0.76	0.61	0.62
Lowland fens	0.08	0.02	0.08	0.42	0.10	0.14

Analysing individual vegetation groups, for six classes within the Helophyte group, the highest mean F1-score was observed for the model based on HS-ALS data, with a value of 0.71, while the lowest was recorded for the model based solely on ALS data, with an F1-score of 0.47 (Fig. 3). The largest differences in F1 values between models were noted for the classes *Typhetum angustifoliae* and *Typhetum latifoliae*, whereas the smallest differences occurred for the class *Phragmitetum*. Within this group of classes, the greatest spatial similarity (Jaccard index equal 0.88) was found between maps generated using ALS-ORTHO-TEX versus ALS-ORTHO data, whereas the lowest similarity was recorded for the pair HS-ALS versus ALS-ORTHO (0.56) (Table 3). It is also noteworthy that in this group, there is no distinct difference in accuracy metrics (F1, Precision, and Recall) between the ALS-ORTHO and ALS-ORTHO-TEX models (Figs. 3, 4, 5).

The six classes belonging to the grassland group generally have relatively low accuracies, with average F1-scores ranging from 0.43 for the ALS scenario to 0.61 for HS-ALS, not exceeding the threshold of 0.8 (Fig. 3). Differences in accuracy for four scenarios within individual classes were relatively small, ranging from 0.14 for ‘other *Molinietalia* communities’ to 0.23 for *Koelerio*-*Corynephoretea*. In terms of accuracy and map similarity (Jaccard index of 0.76), the ALS-ORTHO and ALS-ORTHO-TEX scenarios were the most similar to each other, whereas the greatest dissimilarity was observed between HS-ALS and ALS-ORTHO (Jaccard index of 0.40) (Table 3). Similarly, within the grassland group, minimal differences in accuracy were found between the ALS-ORTHO and ALS-ORTHO-TEX scenarios (Fig. 6).

The four vegetation classes belonging to the tall-herb vegetation group exhibited considerable variability in accuracy, with all F1 values falling below 0.8 (Figs. 3, 6). The highest accuracy metrics were noted for the HS-ALS scenario, with an average F1-score of 0.69, whereas the lowest were observed for the ALS scenario, with a mean F1-score of 0.32; and in this case, none of the classes achieved an F1-score exceeding 0.53. For tall-herb vegetation classes, a positive effect of using HS on model quality is evident. Specifically, the *Cirsium arvense* communities class was practically unrecognisable using datasets other than HS-ALS, with maximum F1-scores not exceeding 0.10 across other scenarios. A similar pattern was observed for the *Filipendulion* class. The result acquired using the HS-ALS scenario was 0.25 higher compared to other scenarios.

In the case of willow thickets, the average accuracies for tree classes were relatively high: the best results were obtained under the HS-ALS scenario (F1 = 0.89), while the lowest were recorded for the ALS scenario (F1 = 0.69) (Figs. 3, 6). For the HS-ALS scenario, F1-scores exceeded 0.8 for all classes, whereas in the other scenarios, this was only true for the class *Salicetum pentandro-cinereae*. The differences between the best and worst results ranged from 0.13 for *Salicetum pentandro-cinereae* to 0.28 for *Salicion albae*. Across all classes and scenarios, relatively high accuracy metrics were achieved, with F1-scores exceeding 0.6 in every case.

Only one class, *Scheuchzerio-Caricetea*, was included in the Lowland fens group. The difference in F1-score between the best-performing scenario (HS-ALS, F1 = 0.74) and the worst (ALS, F1 = 0.63) was 0.11, which is the smallest difference among all vegetation groups (Figs. 3, 6). For this class, the greatest differences in the classified images were observed (based on the Jaccard index), likely due to its very limited representation within the studied area (Table 3). The highest similarity between maps within this group was found between the ALS-ORTHO-TEX and ALS-ORTHO scenarios, with a Jaccard index of 0.41. In contrast, all other scenario pairs exhibited Jaccard coefficients below 0.15, indicating very low spatial congruence.

The macrophyte vegetation was represented by a single class *Potametea, Lemnetea*. The classification accuracies for this class were relatively high across all scenarios, with F1, Recall, and Precision values exceeding 0.7 (Figs. 3, 4, 5, 6). The highest model accuracy was achieved under the HS-ALS scenario (F1 = 0.91), while the lowest was recorded for the ALS scenario (F1 = 0.73). A similarly high accuracy (F1 = 0.90) was also observed for the ALS-ORTHO-TEX scenario. Analysis of map similarity revealed that all maps, regardless of the scenario compared, exhibited high spatial congruence, with similarity indices above 0.7 (Table 3).

In the deciduous and coniferous forest group, five vegetation classes were distinguished. The average difference in F1 accuracy between four scenarios was approximately 0.23, with the highest accuracy achieved under the HS-ALS scenario (F1 = 0.94) and the lowest under the ALS scenario (F1 = 0.70) (Figs. 3, 6). For all classes within this group, the F1-scores were greater than 0.8 under the HS-ALS scenario, whereas only three out of five classes reached this threshold in other scenarios. Notable information regarding this group is the high similarity among the maps, with the similarity coefficient between any two maps consistently exceeding 0.80 (Table 3). The F1 accuracies were very high across all classes in the HS-ALS scenario; however, substantial variability in accuracy was observed between classes in the other scenarios. Specifically, the *Betula* spp. and *Populus tremula* communities exhibited significant accuracy declines, with F1-scores dropping by over 0.20 when classification was performed using scenarios other than HS-ALS.

## Discussion

### Evaluation of dataset utility for vegetation classification

Wall-to-wall vegetation mapping provides extremely valuable information for the management of conservation areas. Such comprehensive information is difficult to obtain for medium and large protected areas based on field mapping methods, in which the inventory is mostly done using Gradient-Oriented Transect Sampling^24^. The disadvantage of the transect methods is that many of the vegetation patches may be overlooked if the interval selected is too large^70^. Remote sensing enables this type of analysis because it captures continuous spatial data regardless of area size. An equally common solution is hybrid mapping, where remote sensing and machine learning are used only in the first stage of vegetation mapping^71^. Recent automated tools, such as the NaturaSat software system, have demonstrated the operational feasibility of integrating satellite-based remote sensing for habitat identification and monitoring, supporting the transition toward wall-to-wall mapping^27^. Nevertheless, limitations related to spatial resolution, phenological dependency, user-driven parameterization, and the transferability of trained models across regions highlight the need for further methodological refinement and systematic validation before such systems can fully replace traditional field-based surveys.

However, even with remote sensing data, obtaining detailed wall-to-wall information is challenging, but the problems lie in other aspects. One such challenge is adapting the spatial resolution and data type to the specifics of the mapped area. In the case of studies conducted in the northern peatlands, it has been proven that reducing spatial resolution significantly reduces mapping accuracy in heterogeneous landscapes^72^. Classifying vegetation using this approach is particularly complex in protected areas, which by definition exhibit high species richness and spatial variation in species composition. This topic was taken up in the case of wetlands, but the number of distinguished classes generally did not exceed twenty. Such analyses have been performed in many areas. For habitat including wetlands mapping of 14 classes on an area of about 48 km^2^^73^, the overall accuracies varied from 0.82 to 0.91, with class accuracies from 0.55 to 1. In the Brière Marshes, where 13 different classes were classified on almost 14 ha, Natura 2000 habitats and other classes had very low out-of-bag estimation error rate between 0.56% and 4.01%^74^. For the coastal wetland vegetation of 5 classes, the OA was from 0.62 to 0.88^75^. The classification was also performed for forest areas. In the Laurentian Mixed Forest on 3151 ha, the classification was performed on the habitat community level, the OA varied from 0.75 to 0.8, whereas the F1 score ranged from 0.60 to 1^76^. Also, 14 different classes from a Mediterranean forest were mapped on 60 km2 with an overall accuracy from 0.7 to 0.82^77^. A maximum of 15 classes are mapped regardless of the article, which helps to achieve higher accuracies. The variety of species and the subtle differences between vegetation types make accurate identification challenging. However, carrying out such detailed vegetation mapping is very valuable because the obtained information can be directly applied in environmental management and conservation efforts. In the performed analysis, 30 different classes were classified, of which 17 classes had an F1 value exceeding 0.75, and the average F1 was equal to 0.76 (Fig. 3). It is also worth noting that 11 of the 30 legend classes have been formulated at the plant community level, i.e. the basic syntaxonomic unit^78^. It can be assumed that the obtained accuracies have similar values ​​to those obtained earlier, while at the same time, with a much larger number of classes. It is worth mentioning that the cited publications most often classified more general vegetation classes, where it is easier to obtain higher accuracy.

A literature review concerning wetland classification indicates that the most effective identification of vegetation classes is achieved through data fusion, particularly the integration of hyperspectral imaging with LiDAR data^13, 72^. Consequently, this approach was adopted as the baseline methodology in the present study^5^. The highest accuracies for each class were obtained using this combination of data, with a mean F1-score of 0.77, compared to 0.52 for LiDAR-only data (Figs. 3, 4, 5). The high spectral resolution of hyperspectral data enables the recognition of plant communities and species, as has been demonstrated previously^32^. Fusion of these datasets yielded the highest accuracies among the analysed data combinations: for 14 classes across the macrophyte vegetation, willow thickets, helophyte communities, and deciduous and coniferous forest groups, F1-scores were equal to or greater than 0.8 (Figs. 3, 4, 5. This approach enables precise delineation of the complex structure of vegetation communities, which is especially critical in wetlands, mountainous, and swampy areas where classification based solely on spectral or structural information may prove insufficient. Studies conducted in diverse wetland ecosystems, including lake environments, have demonstrated the superior performance of hyperspectral data for emergent wetland vegetation classification. Specifically, research at Lake Balaton, Hungary, found that hyperspectral visible and near-infrared data (400–1000 nm provided the most accurate classification results, with the inclusion of LiDAR information not improving classification accuracy^12^. However, recent advances in machine learning methodologies, including adaptive feature enhancement networks (MsAF-EFN) and multisource feature embedding approaches (MsFE-IFN), have successfully optimized the integration of hyperspectral and LiDAR data, demonstrating enhanced classification performance for coastal and karst wetland environments^19–21^. These contrasting findings highlight the context-dependent nature of multisource data fusion, where the effectiveness of integrating hyperspectral and LiDAR data varies across different wetland types and environmental conditions.

The use of LiDAR data alone resulted in an average F1-score of 0.52, which is approximately 0.25 lower than the accuracy achieved with the combined HS-ALS dataset (mean F1 = 0.77). Vegetation mapping based only on LiDAR data is relatively uncommon and generally yields accuracy levels around 60%^79–81^, except in studies where the classification legend complexity is substantially reduced in such cases, accuracies of approximately 0.80 have been reported^15,16^. The results obtained in the present study for the ALS scenario exhibit lower accuracy compared to those documented in the literature. This may be related to the fact that here, vegetation maps were developed with a higher level of legend detail and attempted to discriminate classes exhibiting considerable structural and species composition similarity. Significant variability in accuracy across individual classes is also evident. Nonetheless, LiDAR data are successfully employed for tasks such as tree species identification^82^. Our classification results confirm these observations: the highest accuracy within the ALS scenario was achieved for forest vegetation classes specifically, Riparian/alder forest, Mixed/oak forests, and *Vaccinio-Piceetea* (Figs. 3, 4, 5). Incorporating RGB data with ALS increased overall accuracy by 0.11, raising it to 0.63. For 12 out of 30 classes, accuracy improvements following the addition of RGB data were sufficient to reduce the difference in accuracy between HS-ALS and ALS-ORTHO scenarios to 0.10 or less. This effect was notably observed for classes within the grassland, lowland fens, and macrophyte groups. Similar analyses conducted in mangrove forest areas, where 10 classes were identified, reported LiDAR-only accuracies ranging from 0.86 to 0.89, whereas the fusion of LiDAR with RGB data including spectral bands and texture features yielded accuracies between 0.95 and 0.96^83^.

Numerous studies have employed image data (most commonly RGBN) in combination with LiDAR for vegetation mapping; however, the majority of these investigations utilise UAV platforms^84,85^. Results derived from UAV platforms are, however, more challenging to compare directly to this study because their analyses typically cover significantly smaller areas (e.g., 2 ha –)^85^ and the acquired point cloud density is substantially higher, often around 800 points/m^2^. Consequently, the higher accuracies reported in UAV-based analyses integrating LiDAR and multispectral data are justified. For instance, in monitoring tundra grassland vegetation, accuracies of approximately 0.90 have been achieved^85^. Recent applications of UAV-based RGB imagery combined with deep learning approaches (specifically the Natural Numerical Network, NatNet) for wetland classification and revitalisation monitoring have demonstrated the potential of these platforms for detailed vegetation mapping, achieving classification accuracies of 0.88 and F1 scores of 0.90 in protected wetland areas^26^. However, the operational applicability of such methods remains constrained by the limited spatial and temporal extent of individual studies, as restoration processes are typically spatially limited to smaller areas, which naturally constrains broader generalisation^26^.

The addition of textural layers resulted in an average accuracy increase of approximately 0.02 compared to the ALS-ORTHO scenario. Such a small improvement has also been reported in other studies focused on wetland mapping^32,72^, and the influence of adding textural features varies across individual classes. For example, adding textural data to RGB and DEM datasets for forest and wetland mapping increased OA from 0.73 to 0.79 for the Random Forest classifier and from 0.71 to 0.76 for the Support Vector Machine^76^. In case of our classification, five classes *Potametea*, *Lemnetea*,*Phalaridetumarundinaceae,Typhetumangustifoliae* and *T. latifoliae*; *Betula spp*. communities; and mown or grazed grasses exhibited at least a 0.05 increase in F1-score (Figs. 3, 4, 5). It is also noteworthy that the similarity between resulting maps was greater when comparing ALS-ORTHO-TXT with HS-ALS than when comparing ALS-ORTHO with HS-ALS (Table 3).

Based on the conducted research, it can be concluded that for twelve classes (Sedge swamps; *Phalaridetum arundinaceae*; *Glycerietum maximae*; *Filipendulion*; *Convolvuletalia*; *Calamagrostietum epigeji*; *Cirsium arvense* communities; *Salix rosmarinifolia* communities; *Salicion albae*; *Betula spp*. communities; *Populus tremula* communities; and mown or grazed grasses), the F1 accuracies are at least 10% lower in scenarios that do not include hyperspectral data. This indicates a significant reduction in the usability of vegetation maps when HS data are replaced by RGBN data. The fusion of hyperspectral and ALS data (HS-ALS) constitutes the optimal dataset for wetland management. The effectiveness of hyperspectral and ALS data fusion (HS-ALS) for wetland management varies across different wetland environments and contexts. While research in temperate lake ecosystems has demonstrated that hyperspectral visible and near-infrared data (400–1000 nm) alone provide the most accurate classification results, with the inclusion of LiDAR information not improving classification accuracy^12^, recent advances in machine learning methodologies have successfully optimized HS-ALS integration for coastal and karst wetland environments, achieving enhanced classification performance^19–21^. These contrasting findings highlight the context-dependent nature of multisource data fusion, where the optimal dataset configuration depends on specific wetland characteristics, environmental conditions, and classification objectives^22^. For certain classes including *Potamogetonetea*, *Lemnetea*,*Potentillo-Polygonetalia*,*Nardetalia*,*Vaccinio-Genistetalia*; *Scheuchzerio-Caricetea*; *Typhetum angustifoliae* and *T. latifoliae*; and *Phragmitetum* it is possible to replace hyperspectral data with RGBN data combined with textural layers when hyperspectral data acquisition is not possible (Figs. 3, 4, 5). Although the identification accuracies in these cases do not reach those achieved with HS-ALS data, they are significantly higher (on average by 0.25) compared to the ALS scenario. Moreover, the combination of ALS, orthophotos, and textural data (ALS-ORTHO-TXT) enables the development of a comprehensive vegetation class distribution map.

### The usability of remote sensing maps compared to field mapping

The vegetation classifications conducted using four different remote sensing datasets indicate a significant decline in model accuracy in the absence of HS, which directly impacts the usability of the resulting maps for wetland management. Classifications based on ALS data combined with orthophotos exhibited an overall accuracy reduction of approximately 0.10 compared to the HS-ALS data set, whereas the use of ALS data alone resulted in an accuracy decrease of about 0.20. According to previous studies, an accuracy threshold of 80% is generally considered acceptable to end-users. Only the maps derived from the classification scenario incorporating HS and ALS data surpassed this threshold. The accuracy of other maps ranged from 0.73 to 0.63, indicating partial usability. Despite the considerable accuracy decline for maps generated without hyperspectral data, it is noteworthy that the ALS-ORTHO-TEXT and ALS-ORTHO scenarios still yielded relatively high accuracies, with overall accuracies of 0.73 and 0.72, respectively. These results can be regarded as useful for selected vegetation types and classes exhibiting F1-scores greater than 0.80 (Figs. 3, 4, 5). The full vegetation map may be considered suitable for general purposes, such as overview assessments; however, it is unlikely to meet the requirements for detailed analyses. Vegetation maps produced exclusively from ALS data achieved an overall accuracy of 0.63, which is deemed insufficient for nature conservation purposes. Such maps contain a substantial number of classification errors, with only 5 out of 30 classes attaining F1-scores above 0.80.

**Fig. 7:**
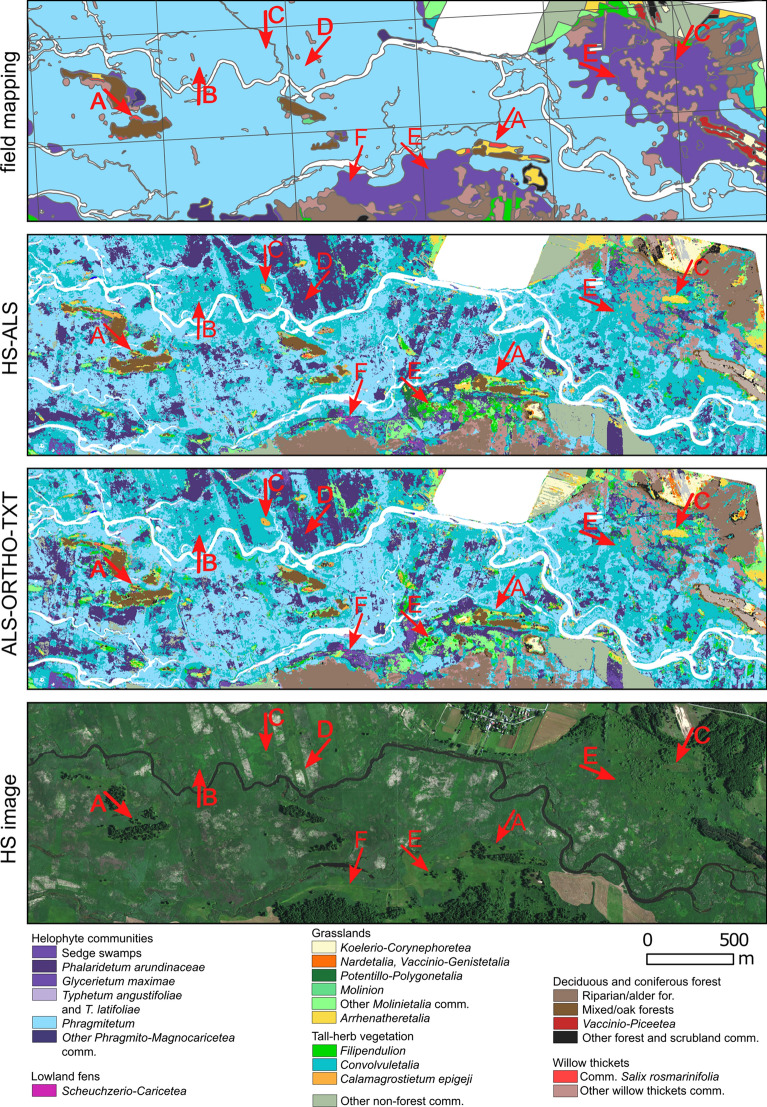
Comparison of vegetation maps based on the field mapping and two remote sensing map based on scenarios with the best average F1 accuracy (HS-ALS and ALS-ORTHO-TXT). In Fig. 1, the red frame indicates the part of the study area for which the map was generated. For comparison purposes, both maps were standardized to a common legend that aggregates certain classes.

An alternative perspective on the results is provided by the comparative analysis of the two best vegetation maps generated using the methods described in this study (scenarios: HS-ALS and ALS-ORTHO-TXT) against a vegetation map produced through traditional field-based phytosociological mapping techniques, which has been previously employed for wetland management within the Park (Fig. 7). This is a vegetation map of the Narew National Park and the PLH200002 Narew Marshes area (Protection Plan for the Narew National Park^86^, unpublished report).

This comparative analysis of maps generated by different methodologies reveals a significant increase in knowledge regarding wetland vegetation facilitated by remote sensing, irrespective of the data source utilised. Accordingly, the results confirm the operational utility of remote sensing methods for monitoring the status of protected areas^87^.

There are clear differences between maps acquired based on field mapping and based on airborne data. On the map acquired based on field mapping are absent many small but ecologically valuable patches, such as meadows developing on mineral soil elevations (Fig. 7, features “C”) and lowland fens (Fig. 7, features “F”). The traditional map presents an overly simplified representation of the extensive mosaic of wetland habitats (Fig. 7, feature “E”). Also, there is no mosaic of interwoven elements of two classes *Phragmiteteum* and *Convolvuletalia* as well as *Phalaridetum arundinaceae* and *Convolvuletalia*, which is difficult to reproduce (Fig. 7, features “B” and “D”). On the other hand, the remote sensing techniques failed to delineate the valuable class *Nardetalia*, which was well represented in the traditional map (Fig. 7, features “A”).

## Conclusions

The comparison of four airborne remote sensing datasets conducted within the Narew National Park for wall-to-wall mapping of vegetation patterns in spatially heterogeneous landscapes enabled the following conclusions to be drawn:

- Replacing hyperspectral data with RGBN data resulted in a decrease in overall accuracy from 0.82 to 0.73. The accuracy decline varied among individual classes, being highest for classes in the tall-herb vegetation group, averaging 0.27, and lowest for classes in the Macrophyte group, averaging 0.01.
- The addition of textural features derived from RGBN data resulted in only a slight improvement in vegetation map accuracy by approximately 0.02.
- Using only LiDAR data caused a significant reduction in overall accuracy by 0.19 compared to the best-performing scenario using combined HS and LiDAR data. The wetland vegetation map produced from the ALS scenario is therefore considered unsuitable for nature conservation purposes.
- Comparing the traditional vegetation map, developed for protected area management within the Park, with remote sensing maps generated through the fusion of LiDAR and image data (HS or RGBN), indicates a substantial increase in information and knowledge regarding the wetland vegetation. The application of remote sensing for vegetation mapping in areas characterised by high spatial heterogeneity is particularly useful and justified.

## Supplementary Information

Supplementary Information.

## Data Availability

The aerial and ground reference data that support the findings of this study are available from Narew National Park but restrictions apply to the availability of these data, which were used under license for the current study, and so are not publicly available. Data are however available from the authors upon reasonable request and with permission of Narew National Park.
