# Supplementary material for: Value of hyperspectral data for wall to wall wetland vegetation mapping in heterogeneous landscapes
Source: Sci Rep. 2026 Mar 17;16:13947. doi: 10.1038/s41598-026-44275-0 (PMC13133186; doi:10.1038/s41598-026-44275-0)
Supplement: Supplementary file 1 — Supplementary Information. [file 41598_2026_44275_MOESM1_ESM.docx]

**Supplemental Material**

**Value of hyperspectral data for wall to wall wetland vegetation mapping in heterogeneous landscapes**

Anna Jarocińska^1^, Dominik Kopeć*^2,3^, Jan Niedzielko^3^, Anna Halladin-Dąbrowska^3^, Marlena Kycko^1^

^1^ University Of Warsaw, Faculty Of Geography and Regional Studies, Krakowskie Przedmieście 30, 00-927 Warsaw, Poland

^2^ University of Lodz, Faculty of Biology and Environmental Protection, Department of Biogeography, Paleoecology and Nature Conservation, Banacha 1/3, 90-237, Łódź, Poland

^3^ MGGP Aero Sp. z o.o., Kaczkowskiego 6, 33-100, Tarnów, Poland

*Corresponding author: Dominik Kopeć, e-mail: dominik.kopec@biol.uni.lodz.pl

The equations of texture features calculated using Simple Haralic Texture Extraction tool (Haralic et al., 1973)

Energy

$$f_{1}=\sum_{i,j} {g(i, j)}^{2}$$

Entropy

$f_{2}=\sum_{i, j} g\left( i, j \right){log}_{2}g(i, j)$ or 0 if $g(i, j)=0$

Correlation

$$f_{3}=\sum_{i, j} \frac{\left( i-\mu\right)\left( j-\mu\right)g(i, j)}{\sigma^{2}}$$

Inverse Difference Moment

$$f_{4}=\sum_{i, j} \frac{1}{1+\left( i-j \right)^{2}}g(i, j)$$

Inertia

$$f_{5}=\sum_{i, j} \left( j-j \right)^{2}g(i, j)$$

Cluster Shade

$$f_{6}=\sum_{i, j} {(\left( i-\mu\right)+\left( j-\mu\right))}^{3}g(i, j)$$

Cluster Prominence

$$f_{7}=\sum_{i, j} {(\left( i-\mu\right)+\left( j-\mu\right))}^{4}g(i, j)$$

Haralick Correlation

$$f_{8}=\frac{\sum_{i, j} \left( i, j \right)g\left( i, j \right)-\mu_{t}^{2}}{\sigma_{t}^{2}}$$

where $\mu_{t}$ and $\sigma_{t}$ are mean and standard deviation of the row or column sums, and $\mu$ is weighted pixel average, $\sigma$ is weighted pixel variance.

### The list of features used in the four classification scenarios

| Nb. | Feature name | Feature information | Source | HS-ALS | ALS | ALS-ORTHO | ALS-ORTHO-TXT |
| --- | --- | --- | --- | --- | --- | --- | --- |
| 1 | MNF Band 1 | MNF transformation | HS | X |  |  |  |
| 2 | MNF Band 2 | MNF transformation | HS | X |  |  |  |
| 3 | MNF Band 3 | MNF transformation | HS | X |  |  |  |
| 4 | MNF Band 4 | MNF transformation | HS | X |  |  |  |
| 5 | MNF Band 5 | MNF transformation | HS | X |  |  |  |
| 6 | MNF Band 6 | MNF transformation | HS | X |  |  |  |
| 7 | MNF Band 7 | MNF transformation | HS | X |  |  |  |
| 8 | MNF Band 8 | MNF transformation | HS | X |  |  |  |
| 9 | MNF Band 9 | MNF transformation | HS | X |  |  |  |
| 10 | MNF Band 10 | MNF transformation | HS | X |  |  |  |
| 11 | MNF Band 11 | MNF transformation | HS | X |  |  |  |
| 12 | MNF Band 12 | MNF transformation | HS | X |  |  |  |
| 13 | MNF Band 13 | MNF transformation | HS | X |  |  |  |
| 14 | MNF Band 14 | MNF transformation | HS | X |  |  |  |
| 15 | MNF Band 15 | MNF transformation | HS | X |  |  |  |
| 16 | MNF Band 16 | MNF transformation | HS | X |  |  |  |
| 17 | MNF Band 17 | MNF transformation | HS | X |  |  |  |
| 18 | MNF Band 18 | MNF transformation | HS | X |  |  |  |
| 19 | MNF Band 19 | MNF transformation | HS | X |  |  |  |
| 20 | MNF Band 20 | MNF transformation | HS | X |  |  |  |
| 21 | MNF Band 21 | MNF transformation | HS | X |  |  |  |
| 22 | MNF Band 22 | MNF transformation | HS | X |  |  |  |
| 23 | MNF Band 23 | MNF transformation | HS | X |  |  |  |
| 24 | MNF Band 24 | MNF transformation | HS | X |  |  |  |
| 25 | MNF Band 25 | MNF transformation | HS | X |  |  |  |
| 26 | MNF Band 26 | MNF transformation | HS | X |  |  |  |
| 27 | MNF Band 27 | MNF transformation | HS | X |  |  |  |
| 28 | MNF Band 28 | MNF transformation | HS | X |  |  |  |
| 29 | MNF Band 29 | MNF transformation | HS | X |  |  |  |
| 30 | MNF Band 30 | MNF transformation | HS | X |  |  |  |
| 31 | ARI1 | Anthocyanin Reflectance Index 1 | HS | X |  |  |  |
| 32 | ARI2 | Anthocyanin Reflectance Index 2 | HS | X |  |  |  |
| 33 | ARVI | Atmospherically Resistant Vegetation Index | HS | X |  |  |  |
| 34 | BAI | Burn Area Index | HS | X |  |  |  |
| 35 | CRI1 | Carotenoid Reflectance Index 1 | HS | X |  |  |  |
| 36 | CRI2 | Carotenoid Reflectance Index 2 | HS | X |  |  |  |
| 37 | CAI | Cellulose Absorption Index | HS | X |  |  |  |
| 38 | CM | Clay Minerals | HS | X |  |  |  |
| 39 | EVI | Enhanced Vegetation Index | HS | X |  |  |  |
| 40 | FM | Ferrous Minerals | HS | X |  |  |  |
| 41 | GARI | Green Atmospherically Resistant Index | HS | X |  |  |  |
| 42 | GCI | Green Chlorophyll Index | HS | X |  |  |  |
| 43 | DGVI | Green Difference Vegetation Index | HS | X |  |  |  |
| 44 | GLI | Green Leaf Index | HS | X |  |  |  |
| 45 | GRVI | Green Ratio Vegetation Index | HS | X |  |  |  |
| 46 | GSAVI | Green Soil Adjusted Vegetation Index | HS | X |  |  |  |
| 47 | GVI | Green Vegetation Index | HS | X |  |  |  |
| 48 | IPVI | Infrared Percentage Vegetation Index | HS | X |  |  |  |
| 49 | IO | Iron Oxide | HS | X |  |  |  |
| 50 | LCAI | Lignin Cellulose Absorption Index | HS | X |  |  |  |
| 51 | MCARII | Modified Chlorophyll Absorption Ratio Index - Improved | HS | X |  |  |  |
| 52 | MNDWI | Modified Normalized Difference Water Index | HS | X |  |  |  |
| 53 | MRENDVI | Modified Red Edge Normalized Difference Vegetation Index | HS | X |  |  |  |
| 54 | MRESI | Modified Red Edge Simple Ratio | HS | X |  |  |  |
| 55 | MSI | Modified Simple Ratio | HS | X |  |  |  |
| 56 | MSAVI2 | Modified Soil Adjusted Vegetation Index 2 | HS | X |  |  |  |
| 57 | MTVII | Modified Triangular Vegetation Index - Improved | HS | X |  |  |  |
| 58 | NLI | Non-Linear Index | HS | X |  |  |  |
| 59 | NBR | Normalized Burn Ratio | HS | X |  |  |  |
| 60 | NDLI | Normalized Difference Lignin Index | HS | X |  |  |  |
| 61 | NDNI | Normalized Difference Nitrogen Index | HS | X |  |  |  |
| 62 | NDWI | Normalized Difference Water Index | HS | X |  |  |  |
| 63 | NMDI | Normalized Multiband Drought Index | HS | X |  |  |  |
| 64 | PRI | Photochemical Reflectance Index | HS | X |  |  |  |
| 65 | PSRI | Plant Senescence Reflectance Index | HS | X |  |  |  |
| 66 | REPI | Red Edge Position Index | HS | X |  |  |  |
| 67 | RGRI | Red Green Ratio Index | HS | X |  |  |  |
| 68 | SAVI | Soil Adjusted Vegetation Index | HS | X |  |  |  |
| 69 | SIPI | Structure Insensitive Pigment Index | HS | X |  |  |  |
| 70 | SGI | Sum Green Index | HS | X |  |  |  |
| 71 | TCARI | Transformed Chlorophyll Absorption Reflectance Index | HS | X |  |  |  |
| 72 | TGI | Triangular Greenness Index | HS | X |  |  |  |
| 73 | TVI | Triangular Vegetation Index | HS | X |  |  |  |
| 74 | VARI | Visible Atmospherically Resistant Index | HS | X |  |  |  |
| 75 | WBI | Water Band Index | HS | X |  |  |  |
| 76 | WVBIU | WorldView Built-Up Index | HS | X |  |  |  |
| 77 | WVIVI | WorldView Improved Vegetative Index | HS | X |  |  |  |
| 78 | WVNII | WorldView New Iron Index | HS | X |  |  |  |
| 79 | WVNHFD | WorldView Non-Homogeneous Feature Difference | HS | X |  |  |  |
| 80 | WVSI | WorldView Soil Index | HS | X |  |  |  |
| 81 | WWWI | WorldView Water Index | HS | X |  |  |  |
| 82 | ARAHBT | All returns above height break threshold (2m) | ALS | X | X | X | X |
| 83 | CC | Canopy Cover | ALS | X | X | X | X |
| 84 | CC_over2m | Canopy Cover over 2m | ALS | X | X | X | X |
| 85 | CHM_1m | Canopy height model | ALS | X | X | X | X |
| 86 | CRS_Intensity | Canopy Reflection Sum (Intensity) | ALS | X | X | X | X |
| 87 | CRS_Reflectance | Canopy Reflection Sum (Reflectance) | ALS | X | X | X | X |
| 88 | deviation_median | Median of devation of pulse shape in grid cell | ALS | X | X | X | X |
| 89 | dq_25 | 25th percentile of devation of pulse shape in grid cell | ALS | X | X | X | X |
| 90 | dq_75 | 75th percentile of devation of pulse shape in grid cell | ALS | X | X | X | X |
| 91 | eigen_largest | Largest eigenvalue of the 3D point distribution | ALS | X | X | X | X |
| 92 | frethmax | Maximum height value for points from first returns | ALS | X | X | X | X |
| 93 | frethmin | Minimum height value for points from first returns | ALS | X | X | X | X |
| 94 | H99_H50 | Heights 99th–50th percentile | ALS | X | X | X | X |
| 95 | HCV | The coefficient of variation of all height points within each pixel | ALS | X | X | X | X |
| 96 | hL2 | Heights second L-moment | ALS | X | X | X | X |
| 97 | HMAD | Median Absolute Deviation of Heights | ALS | X | X | X | X |
| 98 | HmeanThr | Mean height of points above 2m | ALS | X | X | X | X |
| 99 | Htext | Texture of height of points within each pixel, where Htext = St. Dev. (Height > GT and Height < CT) | ALS | X | X | X | X |
| 100 | I50 | 50th Percentile intensity | ALS | X | X | X | X |
| 101 | I75 | 75th Percentile intensity | ALS | X | X | X | X |
| 102 | I99 | 99th Percentile intensity | ALS | X | X | X | X |
| 103 | ikurtosis_Reflectance | Kurtosis of Reflectance | ALS | X | X | X | X |
| 104 | imax_Reflectance | Maximum of Reflectance | ALS | X | X | X | X |
| 105 | imean_Reflectance | Mean of Reflectance | ALS | X | X | X | X |
| 106 | imedian_Reflectance | Median of Reflectance | ALS | X | X | X | X |
| 107 | imin_Reflectance | Minimum of Reflectance | ALS | X | X | X | X |
| 108 | ipcumzq30_Reflectance | Ratio of the sum of Reflectance values computed for points below the 30th height percentile to the sum of Reflectance of all points | ALS | X | X | X | X |
| 109 | ipcumzq50_Reflectance | Ratio of the sum of Reflectance values computed for points below the 50th height percentile to the sum of Reflectance of all points | ALS | X | X | X | X |
| 110 | ipcumzq70_Reflectance | Ratio of the sum of Reflectance values computed for points below the 70th height percentile to the sum of Reflectance of all points | ALS | X | X | X | X |
| 111 | ipcumzq90_Reflectance | Ratio of the sum of Reflectance values computed for points below the 90th height percentile to the sum of Reflectance of all points | ALS | X | X | X | X |
| 112 | ipground_Reflectance | Ratio of the sum of Reflectance values computed for points on ground to the sum of Reflectance of all points | ALS | X | X | X | X |
| 113 | isd_Reflectance | Standard deviation of Reflectance | ALS | X | X | X | X |
| 114 | MeanCanopyH95 | Ratio of the mean height below the 95th height percentile to the number of returns below the 95th height percentile | ALS | X | X | X | X |
| 115 | MeanUnderH | Mean height of points between 0.5m to 90th percentile of height | ALS | X | X | X | X |
| 116 | nG | Number of points below 1m | ALS | X | X | X | X |
| 117 | NMODES | Number of Modes | ALS | X | X | X | X |
| 118 | nonFirst | Ratio of the number of points from the 2nd and 3rd returns to the number of points from the 1st, 2nd and 3rd returns, multiplied by 100 | ALS | X | X | X | X |
| 119 | nongroundStd | Standard deviation of height of points above ground | ALS | X | X | X | X |
| 120 | normalizedShannon | normalized Shannon index | ALS | X | X | X | X |
| 121 | Diffl | Diffl from SAGA tool calculated from DTM | ALS | X | X | X | X |
| 122 | Dirl | Dirl from SAGA tool calculated from DTM | ALS | X | X | X | X |
| 123 | Durl | Durl from SAGA tool calculated from DTM | ALS | X | X | X | X |
| 124 | MCA | MCA from SAGA tool calculated from DTM | ALS | X | X | X | X |
| 125 | MRRTF | MRRTF from SAGA tool calculated from DTM | ALS | X | X | X | X |
| 126 | MRVBF | MRVBF from SAGA tool calculated from DTM | ALS | X | X | X | X |
| 127 | TI | TI from SAGA tool calculated from DTM | ALS | X | X | X | X |
| 128 | TPI | TPI from SAGA tool calculated from DTM | ALS | X | X | X | X |
| 129 | TWI | TWI from SAGA tool calculated from DTM | ALS | X | X | X | X |
| 130 | p4th | Percentage of 4th return points | ALS | X | X | X | X |
| 131 | PARS14 | Ratios of the number of all laser hits within the height strata 8-14 | ALS | X | X | X | X |
| 132 | PARS20 | Ratios of the number of all laser hits within the height strata 14-20 | ALS | X | X | X | X |
| 133 | PARSX | Ratios of the number of all laser hits above 26 | ALS | X | X | X | X |
| 134 | pH2_5 | Percentage of points between 1 to 2.5m | ALS | X | X | X | X |
| 135 | pH10 | Percentage of points between 1 to 10m | ALS | X | X | X | X |
| 136 | ratioMeanSDZ | Ratio of mean vegetation height to standard deviation of vegetation height | ALS | X | X | X | X |
| 137 | SDcanH | Standard deviation of vegetation height from first return | ALS | X | X | X | X |
| 138 | SDmid | Standard deviation of height between zmean -2m to +2m | ALS | X | X | X | X |
| 139 | ShrubDens | Ratio of return number of medium and high vegetation below 3m to all returns | ALS | X | X | X | X |
| 140 | sphericity | Sphericity of points computed from eigenvalues of the points' position covariance matrix: https://github.com/r-lidar/lidR/blob/0a09bcb898ce58634e93b200ea0c6db5345b8ae8/R/metrics_stdmetrics.R#L302 | ALS | X | X | X | X |
| 141 | VDR | Vertical distribution ratio = (P100 – P50)/P100 | ALS | X | X | X | X |
| 142 | zentropy | Entropy of height distribution | ALS | X | X | X | X |
| 143 | zimean_Reflectance | Mean value of the product of height and reflectance | ALS | X | X | X | X |
| 144 | zmax | Maximum height | ALS | X | X | X | X |
| 145 | zpcum3 | Cumulative percentage of returns in the third 2 m thick layer | ALS | X | X | X | X |
| 146 | zpcum4 | Cumulative percentage of returns in the fourth 2 m thick layer | ALS | X | X | X | X |
| 147 | zpcum5 | Cumulative percentage of returns in the fifth 2 m thick layer | ALS | X | X | X | X |
| 148 | zpcum6 | Cumulative percentage of returns in the sixth 2 m thick layer | ALS | X | X | X | X |
| 149 | zq80 | 80th percentile of height distribution | ALS | X | X | X | X |
| 150 | zq90 | 90th percentile of height distribution | ALS | X | X | X | X |
| 151 | zsd | Standard deviation of height distribution | ALS | X | X | X | X |
| 152 | zvar_Reflectance | Ratio of variance of height to Reflectance | ALS | X | X | X | X |
| 153 | Blue band | Blue band of RGBN ortophotomap | ORTHO |  |  | X | X |
| 154 | Green band | Green band of RGBN ortophotomap | ORTHO |  |  | X | X |
| 155 | Red band | Red band of RGBN ortophotomap | ORTHO |  |  | X | X |
| 156 | NIR band | NIR band of RGBN ortophotomap | ORTHO |  |  | X | X |
| 157 | NDVI | Normalised DIfferenve Vegetation index calculated from Red and NIR band of RGBN ortophotomap | ORTHO |  |  | X | X |
| 158 | Blue Energy | texture Energy calculated from Blue band | ORTHO |  |  |  | X |
| 159 | Blue Entropy | texture Entropy calculated from Blue band | ORTHO |  |  |  | X |
| 160 | Blue Correlation | texture Correlation calculated from Blue band | ORTHO |  |  |  | X |
| 161 | Blue Inverse Difference Moment | texture Inverse Difference Moment calculated from Blue band | ORTHO |  |  |  | X |
| 162 | Blue Inertia | texture Inertia calculated from Blue band | ORTHO |  |  |  | X |
| 163 | Blue Cluster Shade | texture Cluster Shade calculated from Blue band | ORTHO |  |  |  | X |
| 164 | Blue Cluster Prominence | texture Cluster Prominence calculated from Blue band | ORTHO |  |  |  | X |
| 165 | Blue Haralick Correlation | texture Haralick Correlation calculated from Blue band | ORTHO |  |  |  | X |
| 166 | NDVI Energy | texture Energy calculated from NDVI band | ORTHO |  |  |  | X |
| 167 | NDVI Entropy | texture Entropy calculated from NDVI band | ORTHO |  |  |  | X |
| 168 | NDVI Correlation | texture Correlation calculated from NDVI band | ORTHO |  |  |  | X |
| 169 | NDVI Inverse Difference Moment | texture Inverse Difference Moment calculated from NDVI band | ORTHO |  |  |  | X |
| 170 | NDVI Inertia | texture Inertia calculated from NDVI band | ORTHO |  |  |  | X |
| 171 | NDVI Cluster Shade | texture Cluster Shade calculated from NDVI band | ORTHO |  |  |  | X |
| 172 | NDVI Cluster Prominence | texture Cluster Prominence calculated from NDVI band | ORTHO |  |  |  | X |
| 173 | NDVI Haralick Correlation | texture Haralick Correlation calculated from NDVI band | ORTHO |  |  |  | X |

### The confusion matrices calculated for each dataset.

###


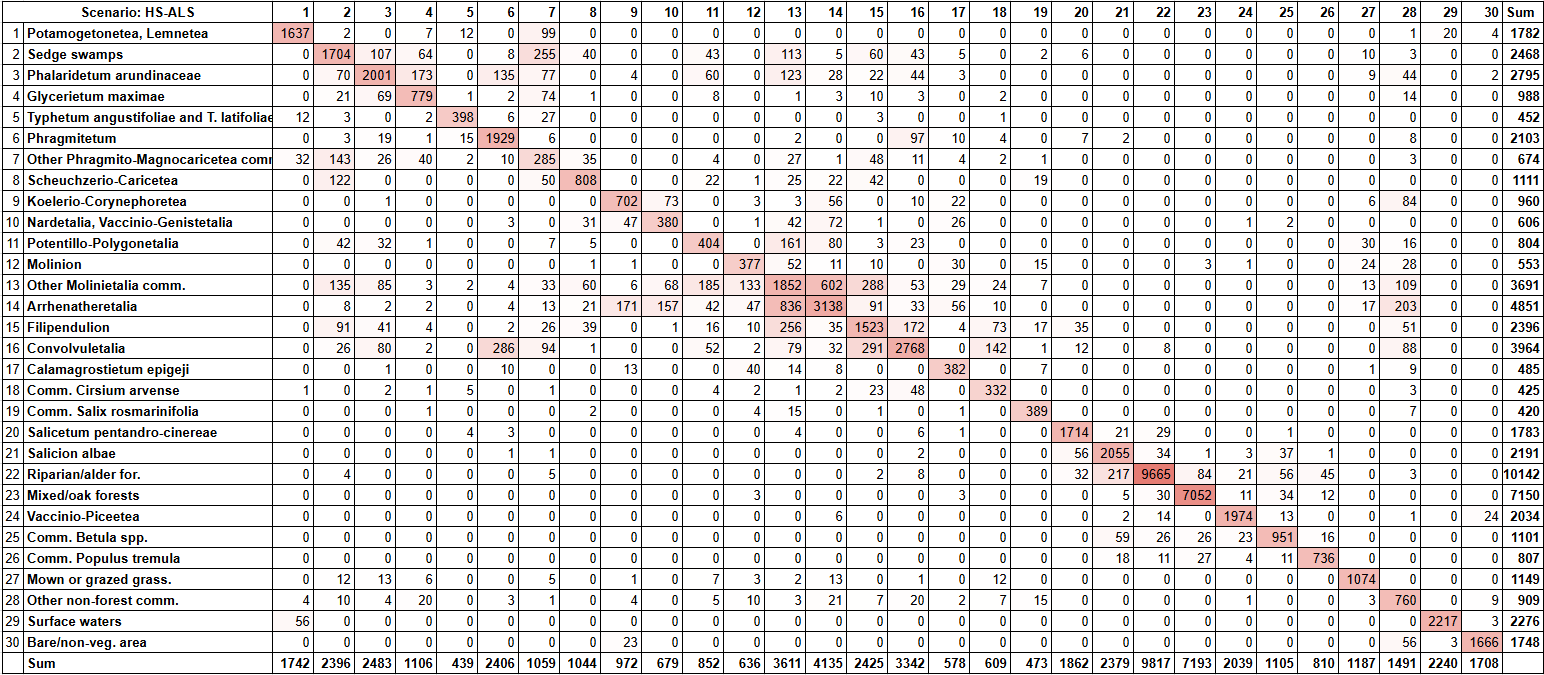


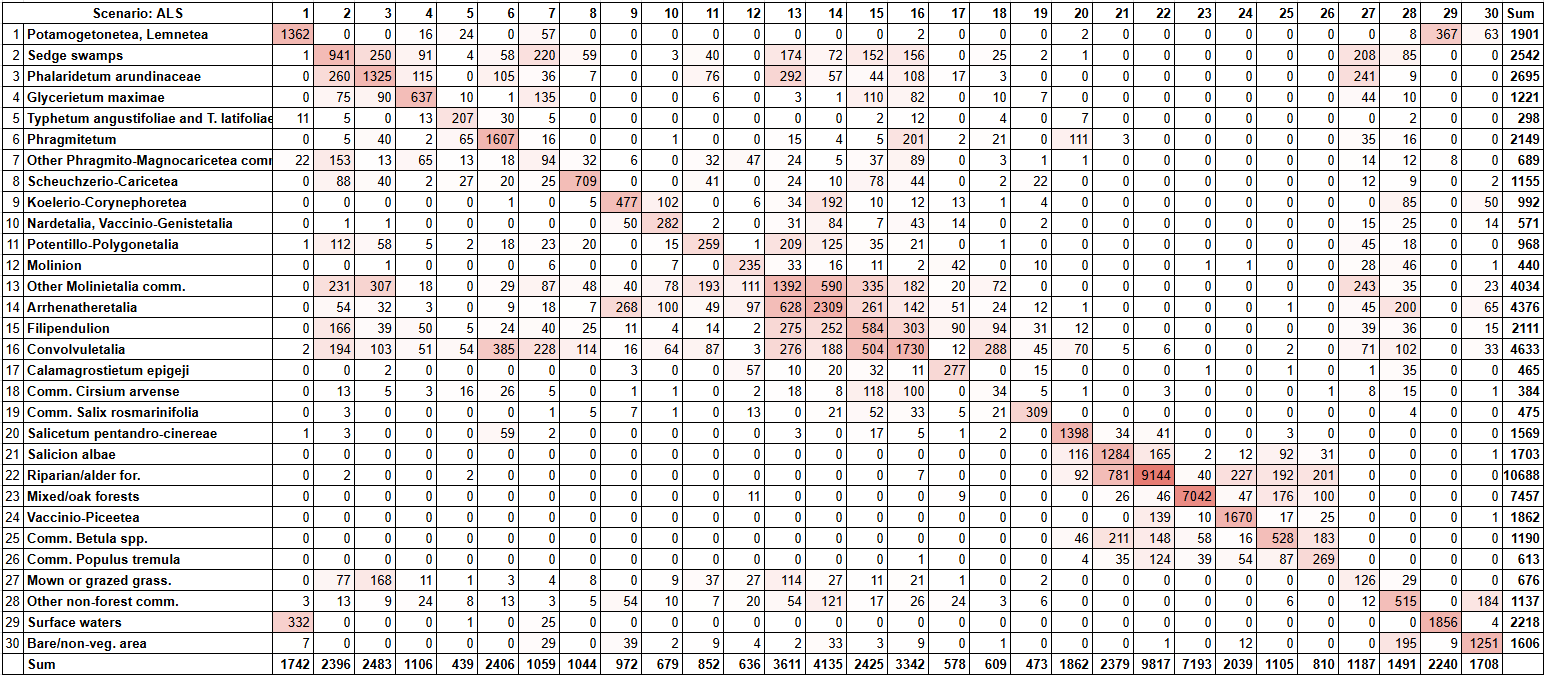


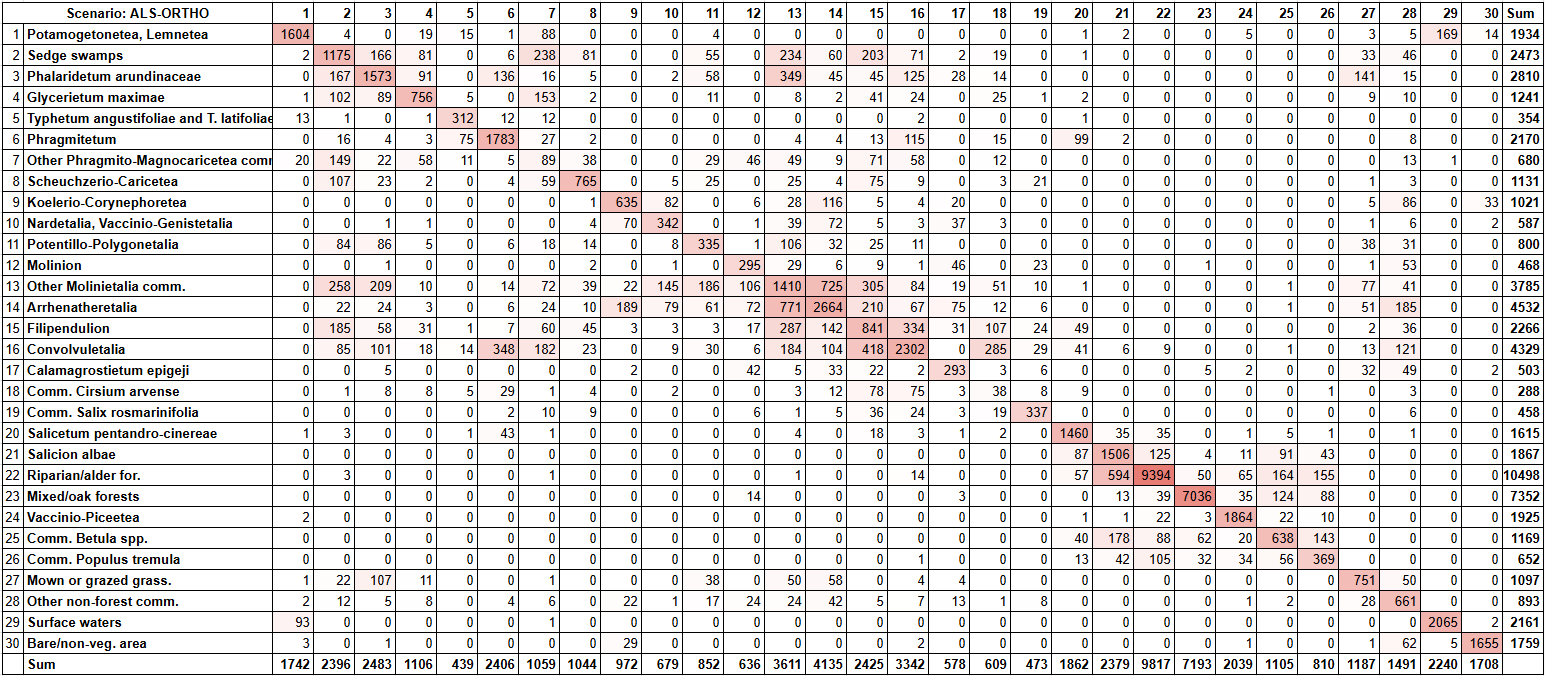


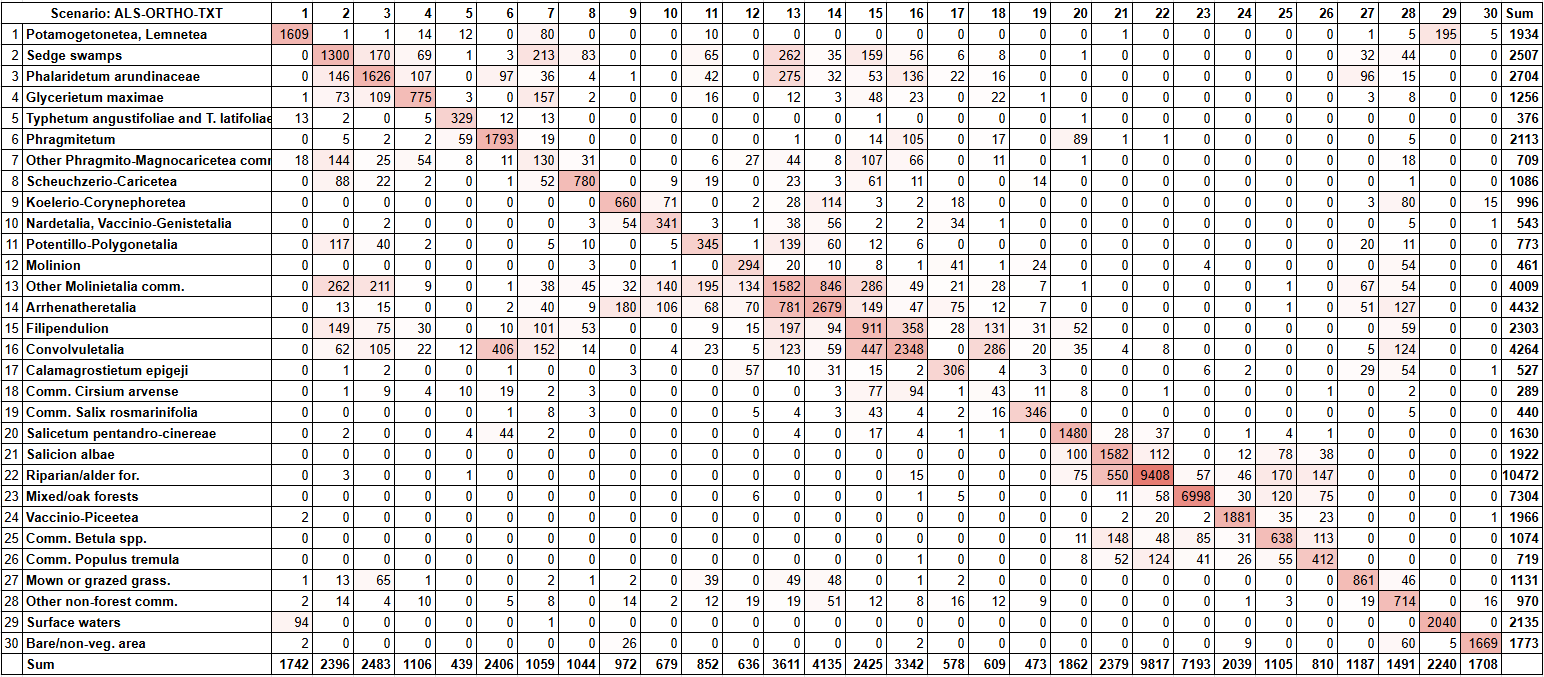


**Reference**

Haralick, R.M., Shanmugam, K., Dinstein, I., 1973. Textural Features for Image Classification. IEEE Trans Syst Man Cybern Part C Appl Rev SMC-3, 610–621. https://doi.org/10.1109/TSMC.1973.4309314
